# P-2185. Community Health Worker-Led Intervention to Increase Hepatitis D Virus Screening among Immigrants in the Metropolitan-DC Area

**DOI:** 10.1093/ofid/ofae631.2339

**Published:** 2025-01-29

**Authors:** Christine Lin, Angeline Nguyen, Chooson Byambaa, Jennifer Galdamez, Hai Chi Le, Sandra Ashford, Lydia Tang

**Affiliations:** University of Maryland School of Medicine, Baltimore, Maryland; Health Betterment Initiative, Washington, District of Columbia; Health Betterment Initiative, Washington, District of Columbia; Health Betterment Initiative, Washington, District of Columbia; Health Betterment Initiative, Washington, District of Columbia; Health Betterment Initiative, Washington, District of Columbia; Institute of Human Virology, University of Maryland school of medicine, Baltimore, Maryland

## Abstract

**Background:**

Hepatitis delta virus (HDV) is the most aggressive form of human viral hepatitis and coinfects people with chronic hepatitis B (HBV), increasing the risk of liver cancer. But HDV screening is not routinely done for people with HBV. In Metropolitan-DC, 5% of Asian, African, and Latino immigrants have HBV (10x the national rate). Therefore, these communities are at risk of HDV and would benefit from increased HDV awareness and screening. Health Betterment Initiative (HBI, an immigrant advocacy group) community health workers (CHWs) routinely assist immigrants with HBV and are well situated to deliver HDV education. However, little is known about the efficacy of CHW-led education interventions in these communities. We seek to evaluate an HDV education intervention aimed at increasing HDV screening in adults with HBV.

**Methods:**

We developed an education intervention with HBI CHWs including educational programming, counseling, and printed materials. The post-intervention group (diagnosed from May 2023-May 2024) received the intervention and were surveyed 3-6 months after to assess HDV screening, knowledge, and usage of materials. We surveyed the pre-intervention group (diagnosed from April 2022-April 2023) to determine baseline HDV screening rate. Our primary outcome will be change in HDV screening from pre- to post-intervention. We present our lessons learnt so far.

**Results:**

Since May 2023, HBI screened 2012 people and diagnosed 22 with HBV. All were from immigrant communities. So far, 18 pre- and 9 post-intervention surveys have been completed. Most were completed in Mongolian and Vietnamese. 20 were born in an Asian country, 6 in an African country, and 1 in El Salvador. Of the pre-intervention group, 4 (22%) reported HDV testing. All in the post-intervention group consulted doctors for HBV, and 1 reported HDV testing. Three recalled receiving our printed materials, but none used them at their doctor’s appointments.

**Conclusion:**

Preliminary findings indicate good linkage-to-care for HBV, but low HDV screening and usage of our educational materials. More work is needed to customize education interventions for immigrant communities. We will use these lessons to consult with stakeholders to refine our intervention to improve engagement.

**Disclosures:**

Lydia Tang, MBChB, Arbutus Biopharma: Grant/Research Support|Gilead Sciences: Grant/Research Support

